# Fire in Hospitals

**Published:** 1909-12-18

**Authors:** 


### FIRE IN HOSPITALS.

In consequence of the great interest shown in this matter by the writers in the "Question Box," we print a summary of the Report which was issued by the Committee appointed by H.R.H. the Prince of Wales as President of King Edward's Hospital Fund to draw up rules for the conduct of the staff in case of fire. The Report first makes it clear that rules must be few, because the buildings and conditions of no two hospitals are alike. In cases of alarm it is important to see that the method of summoning those responsible should avoid startling the patients unnecessarily. The affected area only should be disturbed as far as possible. Every building containing patients should have its own means of informing the fire brigade or police station, which should be used by nurses or others in charge without waiting to see if the local appliances are sufficient. An offer might be made to the Fire Brigade authority to allow a public fire-alarm to be placed at the hospital gate, which would be available alike for the public and the hospital. If the hospital is not on the telephone printed regulations should state where the nearest fire-alarm post can be found and how to use it. If the hospital is on the telephone the London Fire Brigade should be called by telephone and by the fire-alarm post in the street. Near the telephone, if any, the following notice should be placed : "In case of fire ring up in the usual way. Say 'Put me on to the Fire Brigade.' When answered say 'Fire at —— Hospital, —— Street,' and any detail of importance, such as large, small, roof, basement, etc." To stay panic is the first object, once the brigade has been called. This is made difficult in those buildings where alternative staircases are not available, as a *cul de sac* may be formed, particularly on the upper floors. Preventive general measures consist in an adequate supply of "first-aid" apparatus, fire drills quietly arranged at regular intervals, with special regard to the location of risks, for these vary with the structure of each building. Probable places of outbreak can generally be foretold. Those attending the drills should have a special duty, such as the handling of fire appliances, assigned beforehand. If electricity or oil is the cause of fire, sand, wet blanket, or hot water should be used till the engines arrive. When an alarm takes place panic must be prevented, and the place of safety for patients is, as a rule, out of the smoke area. In small buildings outside, in large ones into another block. A wet flannel over the face is useful in a densely smoky atmosphere. It should be well known whose duty it is to remove patients, whose to receive and attend to them, and whose to use the first-aid fire apparatus. This knowledge can only be gained by the drawing up of regulations and by periodic fire drills, which should always refer to the location of risks. In other words, each drill should be directed to the stopping of a fire supposed to have broken out in a definite and likely quarter.

Dr. James Whiteford qualified in 1858, and this year completes 50 years of practice in Greenock. He has been the recipient of various congratulatory addresses, and last week was presented with an address containing 300 signatures and a cheque for £930. Sir Hugh Stewart, Bart., made his presentation on behalf of Dr. Whiteford's patients. The Greenock practitioners have presented their *doyen* with a solid silver salver, a purse of £100, and a congratulatory address. We desire to add our congratulations.

